# Improving Depression Management in Primary Care

**DOI:** 10.1097/CNJ.0000000000001362

**Published:** 2026-03-02

**Authors:** Shayanna Stryd, Nicole Wheeler, Sally Villasenor, Sage Davis

**Affiliations:** **Shayanna Stryd, DNP, RN**, is stepping into the family nurse practitioner role and is dedicated to and passionate about improving the quality and follow-up care for individuals and communities in primary care.; **Nicole Wheeler, DNP, CNM, FACNM, CNE**, is a clinical assistant professor at Wayne State University and is passionate about advancing best practice evidence into clinical practice for underserved and underrepresented populations.; **Sally Villasenor, DNP, RN, ACNP-BC, CNE**, is a clinical assistant professor at Wayne State University and is devoted to improving communication gaps in healthcare and fostering education growth for students and communities.; **Sage Davis, DNP, RN, FNP-C**, is the senior director of clinical performance for Michigan Primary Care Association and is passionate about evidence-based guidelines and recommendations to enhance patient care quality.

**Keywords:** clinical pathway, clinical quality measures (CQMs), depression, integrated care, nursing, PHQ-2, PHQ-9, protocol, quality improvement project

## Abstract

Depression is a common mental illness. Primary care providers are uniquely positioned to screen and manage patients with depression. A clinical pathway protocol may ensure timely screening, diagnosis, and treatment of depression. This evidence-based quality improvement project used an integrated team approach to implement a depression clinical pathway protocol (DCPP) at a multisite faith-based federally qualified health center. Results showed an improvement in one clinical quality measure, screening, and follow-up for depression, whereas center providers reported the protocol saved time and was easy to use. Using a DCPP may bridge the gap in depression screening and follow-up care.

**Figure:**
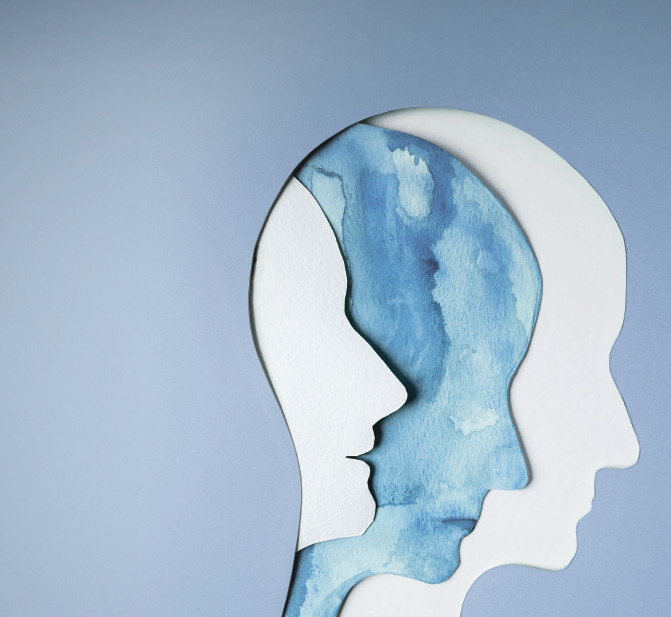
No caption available.

Depression is a common mental illness that affects more than 21 million adults and 3.7 million youth ages 12–17 each year (Mental Health America, n.d.-a). In the United States, 59.8% of youth and 28.2% of adults who have mental illness do not receive any treatment (Mental Health America, n.d.-b). For every 350 individuals, there is only one mental health care provider (National Council for Mental Wellbeing, n.d.). Depression is defined as unhappiness, extreme sadness, or despair that interferes with activities of daily life (American Psychological Association, n.d.). According to the Diagnostic and Statistical Manual of Mental Disorders (DSM-5-TR), major depressive disorder is a mental health condition marked by one or more episodes of at least 2 weeks in which a person experiences a persistently low mood or loss of interest or pleasure in most activities (American Psychiatric Association, 2022). In the primary care setting, 50% of patients are undiagnosed or untreated for depression by their healthcare provider (Siniscalchi et al., 2020).

The U.S. Preventive Services Task Force (2023) recommends annual depression screening with a validated depression screening tool for all adolescents and adults 12 years and up, regardless of risk factors. The Patient Health Questionnaire-2 (PHQ-2) and Patient Health Questionnaire-9 (PHQ-9) are validated depression screening tools (American Psychological Association, 2020). The PHQ-2 is a two-question screening tool that assesses for frequency of depressed mood and anhedonia, quickly identifying individuals who may need further evaluation (Hlynsson & Carlbring, 2024). If the PHQ-2 is positive, the next step is to administer the more comprehensive PHQ-9 to assess if the patient is mildly to severely depressed and may need intervention. If the PHQ-9 is positive, the provider should discuss the need for a treatment plan as well as monitor the patient and repeat the PHQ-9 screen throughout the year until the patient is in remission from depression (Centers for Medicare and Medicaid Services [CMS], 2022). Although standards of care recommend depression screening, many primary care providers lack a clear, comprehensive pathway to ensure alignment with these guidelines.

## PROBLEM DESCRIPTION

Primary care providers can play a crucial role in screening, diagnosing, and treating depression. Approximately 60% of mental health care occurs in the primary care setting, and 79% of antidepressant prescriptions are written by non-mental health providers (Park & Zarate, 2019). Federally qualified health centers (FQHCs) report depression screening performance data through the Health Resources and Services Administration's (HRSA) Uniform Data System (UDS). The Screening for Depression and Follow-Up Plan metric has steadily improved from 64.21% in 2020 to 73.70% in 2024 (HRSA Data Warehouse, 2024). These rates reflect care delivered within FQHCs rather than all U.S. primary care settings. In contrast, national data from Healthy People 2030 (n.d.) indicate that as of 2019, only 9.1% of U.S. primary care office visits included depression screening, with little progress toward the target of 13.5%. Together, these data highlight an ongoing gap in consistent depression screening across broader U.S. primary care.

The current process at our primary care practice is for the medical assistant (MA) to verbally ask the PHQ-2 questions of patients 12 years and older at their annual appointments. If the PHQ-2 score is positive, the MA, registered nurse (RN), or behavioral health provider initiates the PHQ-9. Although our primary care practice had consistently screened for depression, follow-up on positive depression screenings was not standardized.

## AVAILABLE KNOWLEDGE

There has been a considerable amount of research on depression care pathway protocols (DCPP). A pathway protocol ensures continuity of care and timely screening, diagnosis, and treatment using depression practice guidelines for pharmacological and nonpharmacological interventions (Strawbridge et al., 2022). When clinical staff use pathway protocols, complications for patients are reduced and efficacy improves (Lifland et al., 2018). The DCPP reduces delays in care for patients to receive cost-effective, evidence-based high-quality care for depression treatment (Strawbridge et al., 2022).

A DCPP includes screening, diagnosing, and following up with the patient using an integrated team approach to improve care outcomes (Courtney et al., 2024; Dham et al., 2022; Lee et al., 2021; Watson, 2022). The implementation of DCPP has improved earlier rates of routine screenings with the PHQ-2 and PHQ-9 (Aitken et al., 2023; Courtney et al., 2024; Gajaria et al., 2024; Watson, 2022). Several patients who were at risk of depression were not screened until after the DCPP was implemented (Courtney et al., 2022; Dham et al., 2022). Research suggests that approximately one-half of individuals who committed suicide, which is commonly associated with depression, visited their healthcare provider within the previous month (Nock et al., 2022). In one study involving 333,593 patients, suicide attempts declined by 25% in 3 months after depression screening was implemented in primary care (Angerhofer Richards et al., 2024). Clinical staff were able to intervene earlier by assessing for suicide risk and developing safety plans.

In addition to screening, the DCPP saved time and decreased care burden by guiding providers to make treatment decisions (Graham et al., 2020; Khatib et al., 2023). In one study, 94% of providers reported the usefulness of an electronic pathway in saving time, tracking patient trajectories, and enhancing individualized treatment decisions (Hawley et al., 2021). One qualitative study demonstrated that clinical pathways facilitate shared decision-making between adolescents, their caregivers, and providers (Gajaria et al., 2024). Gajaria et al. (2024) also found that the pathway increased the engagement and trust between patients and providers. Fostering engagement and trustworthiness is essential for providers to incorporate into clinical practice so patients feel they are being accurately screened, diagnosed, treated, followed, and feel safe sharing information.

One effective way to improve screening and appropriate treatment for depression is by implementing a DCPP in the primary care setting (Dham et al., 2022). The purpose of this project was to create a DCPP that would standardize the identification and management of depressive symptoms in primary care. Results are reported using the SQUIRE 2.0 guidelines (Ogrinc et al., 2016).

## METHODS

An evidence-based quality improvement project implementing a depression care pathway protocol (DCPP) was conducted at a faith-based federally qualified health center (FQHC) with three locations in Detroit, Michigan. The authors' health center primarily offers comprehensive medical, obstetric, dental, and behavioral health services to uninsured and underinsured individuals, regardless of patient ability to pay. The health center has a mission to share the love of Jesus Christ by providing affordable, integrated quality healthcare to marginalized individuals. The clinic's guiding Bible verse is taken from Matthew 25:36 (NIV), “I was sick and you looked after me,” which reflects the health center's desire to embody Christ's ministry of compassion and service.

To implement the project, we established a core team of stakeholders that included the director of nursing, the behavioral health director, the chief of clinical performance, and the chief medical officer. The stakeholder team's goal was to create a DCPP that could be piloted at the health center's three locations. Stakeholders reached a consensus on the elements of the DCPP, established timelines, and developed strategies to integrate the DCPP into the care process. The DCPP then was shared with medical and behavioral leadership at each of the three clinics. Revisions were made to the DCPP based on feedback from the clinic leadership, and the stakeholder team approved the revised version. Before using the DCPP, the team received approval from the organization's Clinical Governance Committee.

The framework guiding this nurse-driven evidence-based quality improvement project was the *Model for Improvement* (Langley et al., 2009), a widely used established framework that guides organizations in achieving measurable and sustainable improvements in healthcare quality. The model begins by asking three fundamental questions: What are we trying to accomplish? How will we know a change is an improvement? What changes can we make that will result in improvement? (Langley et al., 2009). Once these questions are answered, the model employs the Plan-Do-Study-Act (PDSA) cycle, a structured, iterative process that tests changes on a small scale before implementing the changes more broadly. By using rapid cycles of planning, testing, evaluation, and refinement, organizations can tailor interventions to their local context and build evidence for effectiveness.

### Intervention

Several evidence-based resources were used to develop the DCPP for this project. Kroenke et al. (2001), the original authors of the PHQ-9, provided the foundation for the DCPP. Both the CMS (2022) and Electronic Clinical Quality Improvement Resource Center (2025) use the Preventive Care Screening: Screening for Depression and Follow-Up Plan measure, which was incorporated into this project for its emphasis on annual follow-up for adults and adolescents. The Michigan Community Health Network's (n.d.) Clinical Pathway in Primary Care: Essential Hypertension served as a blueprint for structuring the DCPP. The Children's Hospital of Philadelphia's DCPP for children and adolescents was used to model the layout for the follow-up recommendation steps (Lewis et al., 2023). Finally, recommendations for depression management and follow-up from the American College of Physicians were used to develop the current intervention (Qaseem et al., 2023).

The DCPP includes four steps: screening, diagnosis, treatment, and follow-up. Key roles for each of the steps were assigned to various clinic providers to delineate responsibilities for implementing the DCPP (see Figure 1 as supplemental digital content [SDC] at http://links.lww.com/NCF-JCN/A128). Clinicians used the DCPP following the algorithm, based on their role and the step of the pathway. The algorithm begins with universal screening, where MAs initiate the PHQ-2 for every patient, followed by the PHQ-9 if the initial PHQ-2 screening is positive. Providers then stratify the patient's depression by severity based on the PHQ-9 scores for initial care. For patients with minimal symptoms, monitoring may be sufficient, and treatment may not be necessary. Patients with mild to moderate symptoms may need treatment. Providers were instructed to use clinical judgment to develop a treatment plan. Patients with moderately severe and severe depression warrant active treatment and referral to a behavioral health specialist.

The DCPP also outlines follow-up expectations, including reassessment at defined intervals, documentation of outcomes, and repetition of the PHQ-9 until remission or stabilization is reached. For patients with minimal symptoms, providers would monitor annually and provide education on self-monitoring. For patients with mild or moderate symptoms, providers could consider antidepressant medication and more frequent monitoring (every 1 to 3 months) with a follow-up in 6 months. Patients with moderately severe or severe symptoms received an automatic referral to a behavioral health specialist, antidepressant medication, suicide assessment screening, and safety plan development. Additional resources with embedded links were included at the bottom of the DCPP (see Table 1).

**TABLE 1.** Depression Clinical Pathway Protocol Additional Resources

RESOURCE	LINK
Antidepressant overview	https://www.ncbi.nlm.nih.gov/books/NBK538182/
Medication Adherence Assessment	https://www.stroke.org/-/media/Stroke-Files/Support-Group-Resources/Medication-Adherence-Assessment.pdf
Risk Factors	https://www.aafp.org/pubs/afp/issues/2018/1015/p508.html
Self-Monitor Screening Tool	https://www.cci.health.wa.gov.au/~/media/CCI/Mental-Health-Professionals/Bipolar-Disorder/Bipolar-Disorder---Information-Sheets/Bipolar-Information-Sheet---06---Mood-and-Symptom-Monitoring.pdf
Firearm Safety	https://www.facs.org/media/5kodwasp/firearm-safety-and-patient-health-guide_2022.pdf
Geriatric Depression Scale	https://www.mdcalc.com/calc/10566/geriatric-depression-scale-gds-15
Accurately Diagnosing	https://www.mdcalc.com/calc/10195/dsm-5-criteria-major-depressive-disorder
Safety Planning	https://sprc.org/wp-content/uploads/2023/01/SafetyPlanningGuide-Quick-Guide-for-Clinicians.pdf
Suicide Risk-ASQ Screening Tool	https://www.nimh.nih.gov/sites/default/files/documents/research/research-conducted-at-nimh/asq-toolkit-materials/asq-tool/screening_tool_asq_nimh_toolkit_0.pdf
Support Groups	https://www.nami.org/support-education/support-groups/
Suicide Crisis Line 988	https://988lifeline.org/?utm_source=google&utm_medium=web&utm_campaign=onebox
SDOH Assessment	https://www.cdc.gov/prams/pdf/questionnaire/SDOH-Supplement-Mail-508.pdf
Barriers to Care Screen	https://healthleadsusa.org/wp-content/uploads/2018/10/SDOH-Overcoming-the-Greatest-Barriers-to-Patient-Care.pdf
Eating for Well-being	https://mhanational.org/resources/eating-well-being/
ETOH Screen	https://nida.nih.gov/nidamed-medical-health-professionals/screening-tools-resources/chart-screening-tools
Substance Use Resource	https://www.samhsa.gov/find-help/national-helpline
Exercise Screens	https://www.who.int/docs/default-source/ncds/ncd-surveillance/gpaq-analysis-guide.pdf
Smoking Counseling	https://www.cdc.gov/tobacco/hcp/patient-care/clinical-cessation-tools.html
Self-Help Tools	https://mhanational.org/resources/self-help-tools/
Find a Provider	https://www.zocdoc.com/

The health center's guiding Bible verse, Matthew 25:36, was placed on the DCPP to remind staff of the organization's mission. This message is further reinforced in Matthew 25:40 (NIV): “The King will reply, ‘Truly I tell you, whatever you did for one of the least of these brothers and sisters of mine, you did for me.’” Together, these verses illustrate how caring for the sick is both an act of compassion and a direct expression of faith, embodying Christ's command to serve the most vulnerable.

Education was provided at the monthly virtual staff meeting where each DCPP step was reviewed. An electronic copy of the DCPP and contact information for questions were emailed to all staff (*N* = 47) who provide care at the clinics. Laminated, colored hard copies of the DCPP also were given to each care provider. The project manager met with each provider in person; the teach-back method was used to ensure understanding. The project manager also provided on-site support at each of the three health centers. Concerns could be reported to the project manager during the pilot period.

### Measures

The stakeholders agreed that depression-related CMS clinical quality measures (CQMs) should be used to measure the impact of the DCPP. The CQMs were accessed through a centralized data reporting system, ensuring all patient data were deidentified and anonymous. Data were collected during the implementation phase in May 2024 and compared to data collected prior to the start of the DCPP in January 2024. There was a washout period from February to April while the DCPP was being created.

Seven CQMs were used to measure the impact of the DCPP. These included (1) screening for depression and follow-up; (2) antidepressant medication management-acute phase, which is the initial antidepressant treatment for patients; (3) antidepressant medication management continuation phase, which is assessed with follow-up after the initial antidepressant treatment; (4) depression remission at 12 months, which is when patients are rescreened with the PHQ-9 with a score equal to or less than 5; (5) screening for depression and follow-up plans for 12-17-year-olds; (6) screening for depression and a follow-up plan for 18 years and older; and (7) depression screen positive with follow-up. Due to time constraints of the pilot time frame, depression remission at 12 months was not a 12-month longitudinal collection but a snapshot of patients who had a PHQ-9 score of less than five upon their reassessment. Project data were based on the number of patient encounters that met the established CQM criteria compared to the total number of patient encounters during the project time frame.

In addition to the clinical outcome measures, the project manager developed an anonymous voluntary survey to evaluate the DCPP's use. The survey questions included role description, four yes/no questions, and three open-ended questions (see Figure 2 as SDC at http://links.lww.com/NCF-JCN/A128). The survey was administered on paper and given to all 47 clinic employees (*N* = 47) involved in patient care during their initial meeting with the project manager. Completed surveys (*n* = 17) were collected at the end of the project.

Prior to collecting data, an Institutional Review Board (IRB) Determination Tool was completed; the team determined that, due to the nature of this quality improvement initiative, the DCPP was exempt from IRB oversight. All data were analyzed using descriptive statistics and reported as frequencies and percentages. Responses to the three open-ended questions were used to revise the DCPP after the pilot period.

## RESULTS

The first CQM, screening for depression and follow-up, showed no significant change pre-intervention (*n* = 1847/2068, 89.3%) to post-intervention (*n* = 1436/1611, 89.1%). The second CQM, antidepressant medication management for acute phase treatment, showed a decrease pre-intervention (*n* = 11/35 patients, 31%), compared to post-intervention (*n* = 7/32, 22%). The third CQM, antidepressant medication management continuous phase, showed a decrease pre-intervention (*n* = 8/35, 23%) compared to post-intervention (*n* = 6/32, 19%). The fourth CQM, depression remission at 12 months, showed a decrease pre-intervention (*n* = 1/29, 3.4%) to post-intervention (*n* = 0/20, 0%). The fifth CQM, screening for depression and follow-up plan for 12-17-year-olds, showed an increase pre-intervention (*n* = 35/62, 56.5%), compared to post-intervention (*n* = 31/53, 58.5%). The sixth CQM, screening for depression and follow-up plan for 18 years and older, showed no change in data pre-intervention (*n* = 1229/1357, 91%) compared to post-intervention (*n* = 938/1031, 91%). The seventh CQM, depression screen with positive follow-up, showed an increase pre-intervention (*n* = 10/27, 37%) compared to post-intervention (*n* = 14/16, 87.5%). This change in the seventh CQM was the most significant result that occurred with implementation of the DCPP.

Of the 47 clinic caregivers, 17 reported using the DCPP, including one nurse practitioner (NP), one physician assistant (PA), four physicians (MD), five RNs, five MAs, and one behavioral health specialist (BHS). All respondents (*n* = 17, 100%) reported that the pathway facilitated their depression care flow. Most of the providers (NP, PA, MDs) reported that the DCPP saved time (*n* = 6/7, 86%), whereas 60% (*n* = 6/10) of MAs and RNs found the DCPP to be a time saver. All of NP, PA, and MD providers (*n* = 7) and 90% (*n* = 9/10) of MAs and RNs reported that the DCPP was easy to use. All 17 participants reported that they could see themselves using this pathway in the future.

Responses to the open-ended questions by the 17 participants were summarized into major categories to help with DCPP revisions. The responses to the pros of the DCPP included visual representation of the care flow, delineation of roles, sequential steps clarified, using a multidisciplinary team approach, including resources to aid in care, and streamlining the process for screening and follow-up. The participant responses to the cons of the DCPP included that the pathway was too detailed, providers were not notified when PHQ-2 or PHQ-9s were completed, and difficulty remembering when to rescreen with the PHQ-9 at the next visit within that same year for depression remission.

Respondents made several suggestions for improving the DCPP. Suggestions included using the PHQ-9 at the initial visit instead of the PHQ-2 for a more comprehensive assessment. Another suggestion was to change the color scheme to correspond with the severity of the depression risk score, such as green for 0-4, orange for 5-14, and red for 15-27. Also suggested was extending the first antidepressant medication follow-up appointment from 2 weeks to 4 or 6 weeks to better evaluate medication effectiveness. Other ideas included making the DCPP less visually cluttered and providing a primary care antidepressant education overview for all providers.

## DISCUSSION

The implementation of the DCPP supported the primary purpose of improving depression screening and care, even though not all CQMs for depression improved. One review reported that the number of patients seen for depression after the pathway implementation tripled, emphasizing the increase in undiagnosed patients being treated (Hawley et al., 2021). Based on the results of this project, depression screening with positive follow-up (CQM 7) improved from 37% to 87.5%. Like other results reported in the literature, the DCPP helped improve depression care by reinforcing adherence to timely patient screening and follow-up care (Courtney et al., 2024; Dham et al., 2022; Lee et al., 2021; Watson, 2022). This suggests that the majority of patients who screened positive for depression did not receive follow-up care until after the DCPP was implemented. The DCPP can help the multidisciplinary team adhere to timely screening and follow-up care for individuals who might not otherwise be monitored.

Results from using the DCPP in this project were consistent with other reports where researchers have integrated a multidisciplinary screening and follow-up approach (Courtney et al., 2024; Dham et al., 2022; Lee et al., 2021; Watson, 2022). One strength of the DCPP is the ability to reinforce adherence to timely patient screening and follow-up, as evidenced by the increased positive screening rates after follow-up. Participants who used the DCPP helped adhere to follow-up care for patients who screened positive.

Several factors could have influenced the CQMs that did not improve. Due to the limited time frame of 1 month, not all CQMs had adequate time to improve. In addition, not all staff used the DCPP in their practice during the implementation period. Although the initial intention was for all staff members (*N* = 47) to use the DCPP, only 17 staff participated. The other 30 potential staff members who could have participated may not have done so due to not being present at the initial education session or being unavailable onsite to provide a teach-back. Other barriers could be related to the population served, the limited time frame, and the lack of completed anonymous surveys to confirm the use of the DCPP. There is a possibility that more staff used the DCPP but did not confirm usage by completing the survey.

**FIGURE 3.**
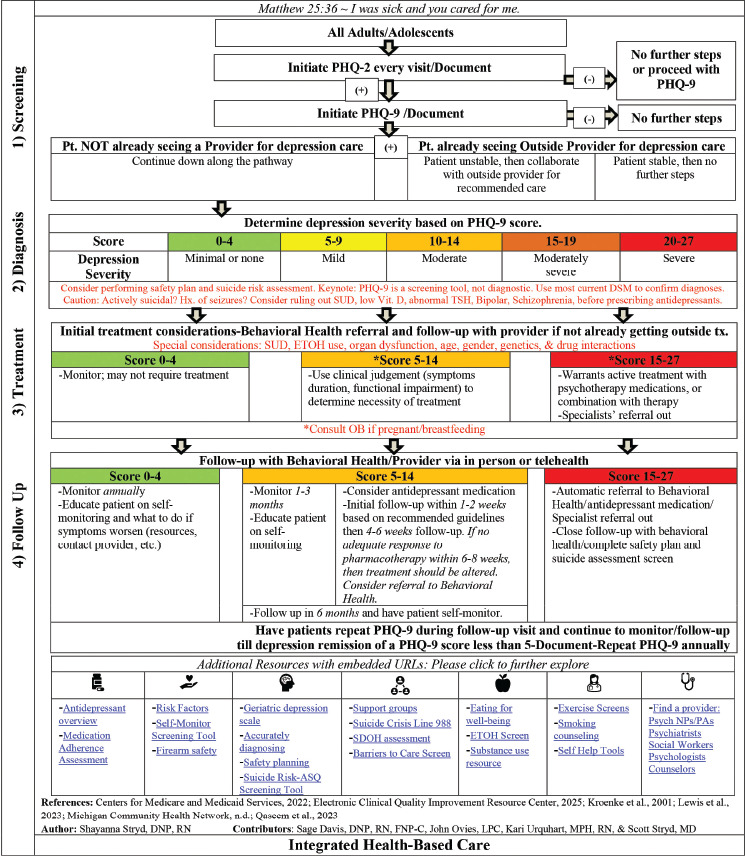
Depression Clinical Pathway Protocol: Final Version

Feedback from staff who participated in the project was one of the most influential key factors in addressing change and ensuring the DCPP's relevance and sustainability. The DCPP was revised based on feedback from participants (Figure 3). The benefit of using the DCPP is the visual step-by-step multidisciplinary process that can help remind providers to prioritize mental health discussions with their patients. Based on participant feedback, the key role pictures were removed to decrease visual clutter. Color coding of the depression severity scores was incorporated as an easy visual element for clinical staff to follow the algorithm.

Some suggestions for change were not implemented. Instead, further education was provided to staff on the rationale for maintaining the original content. For example, one suggestion to change the antidepressant follow-up from 2 weeks to 4-6 weeks to give appropriate time for treatment effects to work (i.e., medication, therapy) was not incorporated based on the clinical guideline from Qaseem et al.'s (2023) recommendation to follow up in 2 weeks. Although antidepressants may not show effects for 4 to 6 weeks, a follow-up appointment is needed before then to ensure the patient is taking the medication consistently and that depression is not worsening.

Other concerns were highlighted, including the lack of notification to providers about completed PHQ-2 and PHQ-9 assessments, the need to rescreen patients when appropriate, and the initial use of either the PHQ-2 or PHQ-9 for screening. Education was provided on how to use the PHQ-2 as a screening tool, followed by the PHQ-9 being used as a more comprehensive assessment for patients who screened positive on the PHQ-2. If clinical staff felt the PHQ-2 did not provide accurate results, an additional option to proceed to the PHQ-9 was incorporated. Additional embedded links to evidence-based resources such as an antidepressant overview also were added.

This quality improvement initiative has limitations that need to be acknowledged. The first limitation was the time frame. Only 1 month of outcomes was measured, which limited the results of this project. Planning for additional time to collect data is needed to better understand the impact of the DCPP. Second, there was inconsistent use of the DCPP because not all staff used the DCPP in practice. Third, the DCPP was used more as a tool than a strict guideline. In the future, an implementation plan should account for consistent use of the DCPP in practice. Consistent use among staff is needed to assess potential DCPP effectiveness at improving depression CQMs.

## CONCLUSION

Acknowledging the importance of mental health by prioritizing mental health discussions with patients can ensure overall well-being. There is a great need for providers at all levels to provide high-quality, timely screening and care, especially for people who may not openly express their mental health concerns. Using a DCPP in clinical practice can bridge the gap in between inadequate and opportune depression care. Incorporating a biblical perspective into the DCPP can enable faith-based health centers to further their mission of sharing the love of Jesus Christ by providing affordable, integrated quality depression care to patients most in need. Matthew 25:40 (NIV), “Truly I tell you, whatever you did for one of the least of these brothers and sisters of mine, you did for me” exemplifies how Jesus has called Christians to live out their faith by caring for the sick. Using a DCPP as an algorithm may help guide clinical staff to meet the individual needs of patients who need depression care. Primary care providers have opportunity to provide compassionate care to patients who are experiencing depression. In doing so, providers reflect the biblical call to “carry each other's burdens, and in this way you will fulfill the law of Christ” (Galatians 6:2, NIV), ensuring that patients not only receive evidence-based treatment but also the compassion and hope that are central to biblically-based care.

**Acknowledgment:** The authors would like to thank everyone who used the Depression Clinical Pathway Protocol in this project.

## Web Resources

- **American Foundation for Suicide Prevention**
https://afsp.org/
- **American Psychiatric Association**
https://www.psychiatry.org/patients-families/depression/what-is-depression
- **Crisis Text Line**
https://www.crisistextline.org/
- **National Alliance on Mental Illness**
https://www.nami.org/About-Mental-Illness/Mental-Health-Conditions/Depression/
- **National Network of Depression Centers**
https://nndc.org/
